# Immediate and one-year outcome of patients presenting with Acute Coronary Syndrome complicated by stroke: Findings from the 2^nd^ Gulf Registry of Acute Coronary Events (Gulf RACE-2)

**DOI:** 10.1186/1471-2261-12-64

**Published:** 2012-08-16

**Authors:** Jassim Al Suwaidi, Khalid Al Habib, Nidal Asaad, Rajvir Singh, Ahmad Hersi, Husam Al Falaeh, Shukri Al Saif, Ahmed Al-Motarreb, Wael Almahmeed, Kadhim Sulaiman, Haitham Amin, Jawad Al-Lawati, Norah Q Al-Sagheer, Alawi A Alsheikh-Ali, Amar M Salam

**Affiliations:** 1 Department of Cardiology, Hamad Medical Corporation (HMC), Doha, Qatar; 2 King Fahad Cardiac Center, King Khalid University Hospital, College of Medicine, Riyadh, Saudi Arabia; 3 Department of Research, Hamad Medical Corporation (HMC), Doha, Qatar; 4 Security Forces Hospital, Riyadh, Saudi Arabia; 5 Saud AlBabtain Cardiac Center, Dammam, Kingdom of Saudi Arabia; 6 Faculty of Medicine, Sana’s University, Sana’a, Yemen; 7 Department of Cardiology, Sheikh Khalifa Medical City, Abu Dhabi, United Arab Emirates; 8 Department of Cardiology, Royal Hospital, Muscat, Oman; 9 Mohammed Bin Khalifa Cardiac Center, Manamah, Bahrain; 10 Department of Non-Communicable Diseases Surveillance and Control, Ministry of Health, Muscat, Oman; 11 Cardiac Center, Al-Thawra Hospital, Sana’a, Yemen; 12 Institute for Clinical Research and Health Policy Studies and Department of Medicine, Tufts Medical Center and Tufts University School of Medicine, Boston, MA, USA; 13 Department of Adult Cardiology, Heart Hospital, Hamad General Hospital (HMC), P.O Box 3050, Doha, Qatar

**Keywords:** Acute coronary syndrome, Myocardial infarction, Stroke, Risk factors, Prognosis

## Abstract

**Background:**

Stroke is a potential complication of acute coronary syndrome (ACS). The aim of this study was to identify the prevalence, risk factors predisposing to stroke, in-hospital and 1-year mortality among patients presenting with ACS in the Middle East.

**Methods:**

For a period of 9 months in 2008 to 2009, 7,930 consecutive ACS patients were enrolled from 65 hospitals in 6 Middle East countries.

**Results:**

The prevalence of in-hospital stroke following ACS was 0.70%. Most cases were ST segment elevation MI-related (STEMI) and ischemic stroke in nature. Patients with in-hospital stroke were 5 years older than patients without stroke and were more likely to have hypertension (66% vs. 47.6%, *P* = 0.001). There were no differences between the two groups in regards to gender, other cardiovascular risk factors, or prior cardiovascular disease. Patients with stroke were more likely to present with atypical symptoms, advanced Killip class and less likely to be treated with evidence-based therapies. Independent predictors of stroke were hypertension, advanced killip class, ACS type –STEMI and cardiogenic shock. Stroke was associated with increased risk of in-hospital (39.3% vs. 4.3%) and one-year mortality (52% vs. 12.3%).

**Conclusion:**

There is low incidence of in-hospital stroke in Middle-Eastern patients presenting with ACS but with very high in-hospital and one-year mortality rates. Stroke patients were less likely to be appropriately treated with evidence-based therapy. Future work should be focused on reducing the risk and improving the outcome of this devastating complication.

## Background

Stroke is the second leading cause of death worldwide and an estimated 795,000 persons have strokes in the USA each year [1]. There appears to be geographic variation in the incidence of stroke and its recurrence even within the same country [2]. The risk of developing stroke as a complication of acute coronary syndrome (ACS) is uncommon, however although this risk is decreasing gradually, the mortality rate from this complication is high [3]. Several studies explored risk factors for stroke after ACS and outcome, however these studies were almost exclusively conducted in developed countries, and data from other ethnicities are lacking [1,3]. We have recently reported that Middle Eastern patients presenting with ACS are relatively younger and are more likely to have diabetes mellitus and metabolic syndrome when compared to their western counterparts [4-6]. Hence, studying stroke in Middle Eastern ACS patients may contribute to our understanding of this complication after ACS. Using data from the First Gulf Registry of Acute Coronary Events (Gulf RACE) we have recently reported low prevalence of stroke associated with *high in-hospital* complication rates among patients presenting acute myocardial infarction [7]. Here, we review the clinical characteristics, in-hospital and 1-year outcome of stroke patients across the whole spectrum of ACS using data from the Gulf RACE-2.

## Methods

### Subjects

The data were collected from a 9-month prospective, multicenter study of the 2^nd^ Gulf Registry of Acute Coronary Events (Gulf RACE) that recruited 7,939 consecutive ACS patients from 6 adjacent Middle Eastern Gulf countries (Bahrain, KSA, Qatar, Oman, United Arab Emirates, and Yemen) between October 2008 and June 2009. Nine subjects were taken out from the study due to non-ACS cases after final diagnosis. Patients diagnosed with ACS, including unstable angina (UA) and non-ST- and ST-elevation myocardial infarction (NSTEMI and STEMI, respectively), were recruited from 65 hospitals. The study was conducted in compliance with the Helsinki Declaration and ethical approval was obtained the Institutional Review Board and Research and Development Committees of the Ministries of Health in Bahrain, Saudi Arabia, Oman, Qatar, United Arab Emirates and Yemen [8].

On-site cardiac catheterization laboratory was available in 43% of the hospitals. There were no exclusion criteria, and thus, all the prospective patients with ACS were enrolled. The study received ethical approval from the institutional ethical bodies in all participating countries [8]. Diagnosis of the different types of ACS and definitions of data variables were based on the American College of Cardiology clinical data standards [9].

A Case Report Form (CRF) for each patient with suspected ACS was filled out upon hospital admission by assigned physicians and/or research assistants working in each hospital using standard definitions and was completed throughout the patient’s hospital stay. All CRFs were verified by a cardiologist then sent on-line to the principal coordinating center, where the forms were further checked for mistakes before submission for final analysis. An enquiry about patients’ survival at 1 and 12 months follow-up after discharged was made.

### Definitions

Stroke was defined as neurologic deficit persisting more than 24 hours. Stroke types; *hemorrhagic* a stroke with documentation on imaging of hemorrhage in the cerebral parenchyma, or subarachnoid hemorrhage, *Ischemic*; a focal neurologic deficit that results from a thrombus or embolus (and not due to hemorrhage) or *Unknown*; if the type of stroke could not be determined by imaging or other means (e.g. from lumbar puncture) or if imaging was not performed [7,10].

### Statistical analysis

Univariate comparisons of patients with and without strokes were made. Continuous variables are presented as means and Standard deviations and compared using the student t tests or Wilcoxon rank sum tests wherever applicable. Categorical variables are shown as percentages, and compared using the Chi-square tests. Bivariate logistics regressions were used taking age, gender, smoking, diabetes mellitus, hypertension, dyslipidemia, systolic blood pressure, heart rate, creatinine, atrial fibrillation, prior MI, prior CABG, peripheral vascular disease, killip class, ACS type, cardiogenic shock, prior aspirin and statin independent variables to see association with in-hospital stroke, in-hospital mortality and one year mortality. Multivariate logistic regressions with forward selection were applied taking significant predictors at p < =0.10 in bivariate analysis for stroke, in-hospital and one year mortality. Unadjusted and adjusted odds ratios (OR) and 95% C.I. were calculated. P value < 0.05 (two tailed) was considered statistically significant at multivariate analysis. Only final multivariate analyses results are provided for in-hospital and one year mortality. Restricted cubic spline functions between SBP and stroke, and SBP and mortality are checked taking quartiles knots of SBP. All analyses were performed using SPSS 18.0 Statistical Package licensed by HMC, Doha, Qatar.

## Results

Of 7,930 patients with ACS in the present analysis, 45.6% had STEMI and 54.4% had non-ST elevation acute coronary syndrome. Fifty-six patients (0.7%) suffered stroke during their index hospitalization. Stroke patients were more likely to present with ST-elevation myocardial infarction (STEMI) (n = 40) and less likely to present with NSTEMI (n = 10) and unstable angina (n = 6). In-hospital stroke was classified as ischemic in 29 patients (52%). Hemorrhagic stroke occurred in 19 patients (34%), while in the remaining 8 patients (14%) stroke type was not identified.

***Baseline characteristics of patients with stroke*** (Table 1).

**Table 1:** Baseline demographics and clinical characteristics for patients with or without prior stroke

**Variable**	**No stroke (n = 7,874 )**	**Stroke (n = 56)**	**P value**
Diagnosis			
STEMI / New or presumed LBBB	45.4%	71.4%	0.001
NSTEMI	30.2%	17.9%	
Unstable Angina	24.4%	10.7%	
Age in years (mean ± SD)	57 ± 12	62 ± 12	0.006
Female gender	21.2%	25%	0.5
Body mass index (kg/m2) (mean ± SD)	27 ± 5	26 ± 4	0.20
Current smokers	52.9%	51.8%	0.9
Previous angina	38.4%	45.3%	0.30
Previous MI	19.8%	20.8%	0.86
Previous PCI	9.2%	5.6%	0.35
Previous CABG	4.3%	3.6%	0.8
Diabetes mellitus*	40.1%	43.6%	0.6
Hypertension†	47.6%	66%	0.01
Hyperlipidemia‡	37.5%	34.8%	0.001
Family history of CAD	11.7%	8.7%	0.53
Previous heart failure	6.7%	13.2%	0.06
Atrial fibrillation	2.1%	7.1%	0.008
Renal failure	4.1%	3.7%	0.89
Peripheral arterial disease	2%	4%	0.26
At presentation			
Presentation >12 hours	58.4%	41.7%	0.1
Ischemic chest pain	84.3%	62.5%	0.001
Atypical chest pain	5%	3.6%	
Dyspnea	7%	10.7%	
Killip Class			0.001
Killip class I	77.2%	53.6%	
Killip class II	14.4%	28.6%	
Killip class III	5.1%	5.4%	
Killip class IV	3.2%	12.5%	
Heart rate (beats/min) (mean ± SD)	84 ± 20	90 ± 19	0.05
Systolic blood pressure (mm Hg) (mean ± SD)	135 ± 29	132 ± 35	0.44
Diastolic blood pressure (mm Hg) (mean ± SD)	81 ± 17	90 ± 19	0.05
GRACE risk score			
Low	39%	20%	0.001
Intermediate	39%	36%	
High	22%	44%	
Medications before admission			
Aspirin	40.5%	43.6%	0.4
Clopidogrel	12.4%	8.9%	0.4
Β-blockers	29.1%	25%	0.5
ACE inhibitors	25.6%	33.9%	0.2
Angiotensin-receptor blockers	4.7%	1.8%	0.3
HMG-CoA reductase inhibitor	31.5%	30.4%	0.9
Laboratory investigations			
Creatinine	101 ± 74	140 ± 124	0.03

Data are expressed as mean or as number (percentage).

* Patient had been informed of the diagnosis by a physician before admission and was undergoing treatment for type 1 or 2 diabetes.

† Systolic blood pressure >140 mm Hg, diastolic blood pressure >90 mm Hg, or current antihypertensive treatment.

ACE-inhibitors = angiotensin-converting enzyme inhibitors.

‡ Total cholesterol >6.1 mmol/L, low-density lipoprotein >4.1 mmol/L, or high-density lipoprotein <0.9 mmol/L; triglycerides >200 mg/dl; or current use of lipid-lowering agent; MI = myocardial infarction; PCI = percutaneous coronary intervention; CAD = coronary artery diseases; CABG = Coronary Artery Bypass Graft.

Patients who suffered in-hospital stroke were about 5 years older than patients without stroke (62 ± 12 vs. 57 ± 12, *P* =0.006). Stroke patients were less likely to have dyslipidemia (34.8% vs. 37.5%, *P* = 0.001) but were more likely to have hypertension (66% vs. 47.6%, *P* = 0.01) and atrial fibrillation (7.1% vs. 2.1%, *P* = 0.008). Stroke patients were more likely and to present with atypical cardiac symptoms , advanced Killip class II-IV (*P* = 0.001), higher heart rate (90 vs. 84 beat per minute, *P* = 0.05), diastolic blood pressure (90 ± 19 vs. 81 ± 17 mmHg; *P* = 0.05) and GRACE risk scoring. There were no significant differences between the two groups in regards to the prevalence of diabetes mellitus, smoking, previous cardiovascular disease, renal failure, peripheral arterial disease or systolic blood pressure. Prior aspirin, clopidogrel, Β-blockers, vasodilators and statins were comparable between the 2 groups. Serum creatinine was significantly elevated among stroke when compared to non-stroke patients.

***In-hospital Treatment patterns of patients with stroke*** (Table 2).

**Table 2:** In-hospital therapy and at discharge in patients with or without stroke

	**During admission**			**At discharge**		
	**No stroke**	**Stroke**	**P value**	**No stroke**	**Stroke**	**P value**
	**(n = 7,874)**	**(n = 56)**		**(n = 78744)**	**(n = 56)**	
Coronary angiography	32.6%	12.5%*	0.002			
Percutaneous coronary intervention	14.5%	5.4%	0.08			
Elective	10.2%	1.8%	0.42			
Urgent/Emergency PCI (UA/NSTEMI)	4.2%	3.6%				
Aspirin	98%	91%	0.001	93%	57%	0.001
β blockers	75%	46%	0.001	80%	38%	0.001
Clopidogrel	76%	55%	0.001	68%	38%	0.001
ACE inhibitors	25.6%	33.9%	0.16	72%	47%	0.001
Angiotensin receptor blockers	4.7%	1.8%	0.30	6.8%	1.8%	0.1
HMG-CoA reductase inhibitor	95%	89%	0.06	92%	58%	0.001
GPIIb/IIa inhibitors	7.8%	3.6%	0.25			
Thrombolytic therapy						
t-PA	3%	13%	0.09			
Streoptkinase	42%	40%				
Reteplase	44%	47%				
Tenektopase	11%	0%				
Unfractionated heparin	49.5%	33.9%	0.02			
LMWH (enoxaparin)	39.1%	21.4%	0.02			

Data are expressed as percentage; STEMI = ST elevation myocardial infarction; NSTEMI = non ST elevation myocardial infarction; UA = unstable angina; LBBB = left bundle branch block; PCI = percutaneous coronary intervention; LVEF = left ventricular ejection fraction.

The treatment patterns for patients with stroke are presented in Table 2. Stroke patients were less likely to be treated with aspirin, clopidogrel, unfractionated heparin, low molecular weight heparin, glycoprotein IIb/IIIa inhibitors or thrombolytic therapy. Overall use of coronary angiography was low in our registry and its use was even lower among patients who had stroke (12.5% vs. 32.6%). Similar results were found in elective (1.8% vs. 10.2%) and emergency (3.6% vs. 4.2%) percutaneous coronary interventions (PCI). Stroke patients who were discharged alive were less likely to be prescribed aspirin, clopidogrel, Β-blockers, ACE inhibitors and statins at discharge.

**Figure 1:**
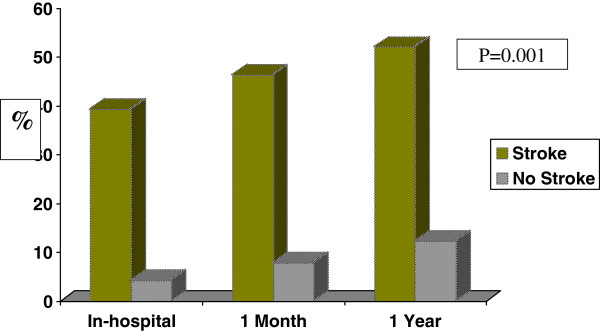
In-hospital, one month and one year mortality rate according to the presence and absence of stroke among acute coronary syndrome patients.

***In-hospital and One-year outcomes of patients with stroke*** (Table 3, Figure 1).

**Table 3:** In-hospital and one-year outcomes of patients with or without stroke ACS patients

**Variable**	**No stroke (n = 7,874 )**	**Stroke (n = 56)**	**P value**
Hospital outcome			
Recurrent ischemia	15%	32%	0.001
Reinfarction	2%	10.7%	0.001
Congestive heart failure	12.9%	39.3%	0.001
Cardiogenic shock	34%	5.6%	0.001
Major bleeding	0.5%	12.5%	0.001
Predischarge echocardiogram (LVEF < 30)	76.5%	71.4%	0.37
Hospital stay (days)	5.9 ± 6	13.45 ± 14	0.001
Mortality			
In-hospital	4.3%	39.3%	0.001
30 day	7.9%	46.3%	0.001
One year	12.3%	52%	0.001

Data are expressed as percentage; STEMI = ST elevation myocardial infarction; NSTEMI = non-ST elevation myocardial infarction; UA = unstable angina; LBBB = left bundle branch block; PCI = percutaneous coronary intervention; LVEF = left ventricular ejection fraction.

Patients who suffered in-hospital stroke were more likely to have their hospital course complicated with recurrent myocardial ischemia (32% vs. 15%), recurrent myocardial infarction (10.7% vs. 2%), cardiogenic shock (34% vs. 5.6%) and major bleeding (12.5% vs. 0.5%) compared to those without stroke. Death occurred in 39.3% of patients who suffered in-hospital stroke compared to 4.3% of those who did not. Length of stay was significantly longer among stroke compared to non-stroke patients (13.5 vs. 5.9 days; *P* = 0.001).

The mortality rate at one month (46.3% vs. 7.9%; *P* = 0.001) and one year (52% vs. 12.3%; *P* = 0.001) were significantly higher among stroke patients when compared to non-stroke patients.

***Stroke Subtypes*** (Table 4).

**Table 4:** Clinical Characteristics and therapy according to stroke subtypes

	**Ischemic**	**Hemorrhagic**	**Not identified**	**P-value**
No (%)	29(52%)	19 (34%)	8(14%)	
Age	60.5 ± 12	60 ± 11	67 ± 15	0.41
Gender Female	10(34.5)	2(10.5)	2(25)	0.17
Diabetes Mellitus	16(52.2)	7(36.8)	1(12.5)	0.08
Hypertension	20(69)	9(47.4)	6(75)	0.23
Atrial fibrillation	4(13.8)	0(0)	0(0)	0.14
Heart rate	92 ± 19	89.9 ± 15	83 ± 30	0.54
Systolic blood pressure	132 ± 30	139 ± 35	116 ± 50	0.29
Diastolic blood pressure	76 ± 24	84 ± 19	68 ± 32	0.28
STEMI	20(69)	16(84.2)	4(50)	
NSTEMI	6(20.7)	1(5.3)	3(37.5)	
UA	3(0.3)	2(10.5)	1(12.5)	0.34
Coronary Angiogram	5(17.2)	1(5.3)	1(12.5)	0.30
PCI	2(6.9)	1(5.3)	0(0)	0.47
Medications on admission				
Thrombolytic therapy	10(52.6)	5(31.2)	0(0)	0.11
Glycoprotein IIb/III inhibitors	12(41.4)	7(36.8)	6(75)	0.17
aspirin	26(89.7)	17(89.5)	8(100)	0.63
Clopidogrel	15(51.7)	6(31.6)	4(50)	0.37

Subset analysis revealed no significant differences between the 3 stroke subtypes in regards to baseline clinical characteristics, MI types or therapies, although there was a trend of increased hemorrhagic stroke among patients treated with thrombolytic therapy.

***Multivariate predictors of stroke*** (Table 5).

**Table 5:** Bivariate and multivariate logistic regression with forward selection for in-hospital stroke patients with acute coronary syndrome

**Variables**	**Unadjusted OR**	**95% C.I.**	**Adjusted OR**	**95% C.I.**
Age	1.03	1.01 – 1.05	--	--
Gender Female	1.24	0.67 – 2.27	--	--
Smoking	0.78	0.44 – 1.39	--	--
Diabetes mellitus	1.16	0.68 – 1.97	--	--
Hypertension	1.87	1.09 – 3.22	2.75	1.50 – 5.26
Dyslipidemia	0.89	0.50 – 1.6	--	--
Systolic Blood Pressure				
<=120 mmgh	1			
121-150 mmgh	1.22	0.66 – 2.26	--	--
> = 151 mmgh	1.09	0.54 – 2.22	--	--
Heart rate (beets /minute)	1.01	1.00 – 1.02	--	--
Creatinine (mg/dl)	1.06	0.69 – 0.84	--	--
Atrial Fibrillation	3.70	1.30 – 10.0	--	--
Prior MI	1.06	0.50 – 2.01	--	--
Prior CABG	0.83	0.20 – 3.40	--	--
Peripheral Vascular Disease	2.00	0.50 – 8.00	--	--
Killip Class > 1	2.95	1.73 – 5.00	1.90	1.03 – 3.50
ACS type Stemi/LBBB	3.00	1.68 – 5.38	2.94	1.56 – 5.52
Cardiogenic Shock	9.15	5.19 – 16.12	5.72	2.96 – 11.04
Prior Aspirin	1.30	0.78 – 2.28	--	--
Statin within 24 hrs	0.50	0.20 – 1.07	--	--

Bivariate results showed age (OR = 1.03, 95% C.I.:1.01-1.05); hypertension (OR = 1.87, 95% C.I.: 1.09-3.22); Atrial fibrillation (OR = 3.70, 95% C.I.:1.30-10.0); advanced killip class >1 (OR = 2.95, 95% C.I.: 1.73-5.0); ACS type STEMI (OR = 3, 95% C.I.: 1.68-5.38); and cardiogenic shock (OR = 9.15, 95% C.I: 5.19-16.12) significant variables whereas; advanced killip class, history of hypertension, STEMI and cardiogenic shock were found associated with increased risk of in-hospital stroke in multivariate analysis.

***Multivariate predictors of in-hospital death*** (Table 6).

**Table 6:** Predictors of in-hospital mortality in ACS patients according to Multivariate logistic regression with forward selection

**Variables**	**In-hospital mortality**	
	**Adjusted OR**	**95% C.I.**
Creatinine (mg/dl)	1.47	1.27 – 1.70
Cardiogenic Shock	85.48	34.5 – 212.0
Stroke	41.75	4.09 – 426.7

All Independent Variables having p < =0.10 at bivariate analysis for in-hospital mortality were considered for multivariate analysis. In-hospital stroke was found independently associated with a significantly higher risk of in-hospital death. The odds of mortality was 42 times higher with 95% C.I. (4.09 – 426.7) in patients with stroke compared to those without stroke.

***Multivariate predictors of one-year mortality*** (Table 7).

**Table 7:** Predictors of one year mortality according to Multivariate logistic regression with forward selection in ACS patients

**Variables**	**One year mortality**	
	**Adjusted OR**	**95% C.I.**
Age	1.03	1.01 – 1.05
Diabetes Mellitus	1.64	1.05 – 2.56
Creatinine (mg/dl)	1.50	1.25 – 1.82
Atrial Fibrillation	5.01	1.09 – 23.1
Killip Class >1	1.97	1.22 – 3.18
Cardiogenic Shock	8.08	4.09 – 15.9
Stroke	12.53	1.91 – 81.9

At one year also stroke was found independently associated with a significantly higher risk of mortality having OR = 13.0 and 95% C.I (1.91-81.9) in stroke patients in comparison to non-stroke.

## Discussion

The current study reports low prevalence of stroke among Middle Eastern patients presenting with ACS. 52% of these strokes were ischemic in origin and 14% were hemorrhagic. Risk factors of stroke were older age, STEMI presentation, atrial fibrillation and history of hypertension. Stroke patients were less likely to be appropriately treated with evidence-based therapy during hospitalization and at discharge. Although the prevalence of stroke was low and comparable to that of reported registries in Western countries, the consequences among stroke patients were dismal with 46% in-hospital mortality and 52% one-year mortality rates.

The reported prevalence of stroke from studies performed mainly in the Western world varied between as low as 0.31% to 1.9% [9-30]. The advent of thrombolysis and primary PCI undoubtedly resulted in significant reduction in stroke risk from 5% in the 1970s to 1% in the current era. The current study reports 0.7% stroke prevalence among Middle Eastern patients presenting with ACS patients in the current era which consistent with previous reports. On the other hand, Ng et al. [19] reported a 7.2% one-year risk of stroke among small population of Chinese MI patients, which is much higher than other reports including ours from a previous registry [7]. When compared to the current study, stroke patients in the National Registry of Myocardial Infarction (NRMI) 3&4 and the Wercester Heart Attack Study were significantly older (>10 yrs) and less likely to have diabetes mellitus [3,17]. Also, while thrombolysis is the main modality of reperfusion therapy in the current registry, significantly more patients received primary PCI in the other studies. The current study suggests that the reduction in stroke risk most likely related to the overall improvement in health care including the provision of evidence-based therapy regardless of the primary reperfusion therapy.

Older age, systemic hypertension, dyslipidemia and ST-elevation myocardial infarction were independent risk factors of stroke in our registry. This is consistent with some of the previous reports. Other independent risk factors previously reported in other reports include prior or in-hospital CABG, renal impairment, low body weight and elevated admission heart rate [11-32]. These variability in findings may be attributed to several factors including patients’ population, ethnicities, use of thrombolytic therapy versus primary percuatenous revascularization therapy and underscore the need of further international studies that include adequate representations of female gender and various ethnicities [33]. There were no significant differences between patients according to their stroke subtypes in regards to clinical characterstics, MI types or therapies although there a trend of higher use of thrombolytic therapy among hemorrhagic stroke patients.

We observed 39.3% in-hospital mortality among stroke patients, which is almost 10 times higher than ACS who did not develop stroke. This suggests that although therapeutic advancement in the management of ACS patients has resulted in remarkable improvement in outcome, this improvement in outcome unfortunately was not observed among stroke patients, this underscores the urgent need to study ways to improve this outcome. This is consistent with pervious reports from the developed world, which observed a mortality rate from ischemic stroke of up to 10-40%, and even higher with hemorrhagic stroke. Stroke patients among our patients population were less likely to be treated with evidence-based therapy at admission and on discharge, which may contribute to the high mortality rate at one-year follow-up. These observations are consistent with that reported by Lee TC et al. [33].

### Limitations

Our data were collected from an observational study. The fundamental limitations of observational studies cannot be eliminated because of the nonrandomized nature and unmeasured confounding factors. However, well-designed observational studies provide valid results and do not systemically overestimate the results compared with the results of randomized controlled trials. We did not look for risk factors by day of onset. Stroke occurring within first few days may be different from those occurring a week or two later.

## Conclusion

The current study reports low prevalence of stroke among Middle Eastern ACS patients with very high in-hospital mortality rate. While older age, anterior MI, hypertension and dyslipidemia at admission were associated with increased risk. Future work should be focused on reducing the risk and outcome of this devastating complication.

## Abbreviations

ACS: Acute coronary syndrome; Gulf RACE: Gulf Registry of Acute Coronary Syndrome; GRACE: Global Registry of Acute Coronary Events; STEMI: ST elevation myocardial infarction; NSTEMI: Non-ST elevation myocardial infarction; CRF: Case report form; ACE inhibitors: Angiotensin converting enzyme inhibitors; PCI: Percutaneous coronary intervention; CABG: Coronary artery bypass grafting; NRMI: National Registry of Myocardial Infarction.

## Competing interests

The authors declare that they have no competing interests.

## Authors’ contributions

JA, KA, NA- participated in the design of the study, patients’ recruitment, writing, analyzing and reviewing the paper. RS- performed the statistical analysis. AH, HAF, SAS, WA, HA, JAL, NQA, AAA All-participated in the patients recruitement, analyzing and reviewing the manuscript. All authors read and approved the final manuscript.

## Funding

Gulf RACE is a Gulf Heart Association (GHA) project and was financially supported by *Sanofi Aventis*, the GHA, Medical Research Center, Hamad Medical Corporation and the College of Medicine Research Center at King Khalid University Hospital, King Saud University, Riyadh, Saudi Arabia. The sponsors had no role in study design, data collection, data analysis, writing of the report, or submission of the manuscript. The study obtained ethical approvals prior to the study.

## Pre-publication history

The pre-publication history for this paper can be accessed here:

http://www.biomedcentral.com/1471-2261/12/64/prepub
